# Small Non-coding RNAs: Do They Encode Answers for Controlling SARS-CoV-2 in the Future?

**DOI:** 10.3389/fmicb.2020.571553

**Published:** 2020-09-18

**Authors:** Pallabi Bhattacharyya, Subhas C. Biswas

**Affiliations:** Cell Biology & Physiology Division, CSIR-Indian Institute of Chemical Biology, Kolkata, India

**Keywords:** COVID-19, coronaviruses, RNA viruses, microRNAs, non-coding RNAs, MERS-CoV, SARS-CoV-2, SARS-CoV

## Abstract

SARS-CoV-2 (severe acute respiratory syndrome coronavirus 2) is a novel coronavirus responsible for the current COVID-19 (coronavirus disease 2019) pandemic, which has hit the world since December 2019. It has spread to about 216 countries worldwide, affecting more than 21.7 million people so far. Although clinical trials of a number of promising antiviral drugs and vaccines against COVID-19 are underway, it is hard to predict how successful these drug- or vaccine-based therapeutics are eventually going to be in combating COVID-19 because most of such therapeutic strategies have failed against human coronaviruses such as SARS-CoV and MERS-CoV (Middle East respiratory syndrome coronavirus) responsible for similar pandemics in the past. In that context, we would like to bring to scientific attention another group of endogenous regulatory molecules, the small non-coding RNAs, especially the microRNAs, which are found to regulate critical cellular pathways in a number of disease conditions, including RNA viral infections. This review will focus on understanding the effect of altered microRNA expression during coronavirus-mediated infections and how it may provide clues for further exploring the pathogenesis of SARS-CoV-2, with a view of developing RNAi-based therapeutics and biomarkers against COVID-19.

## Introduction

Severe acute respiratory syndrome coronavirus 2 (SARS-CoV-2) or human coronavirus 2019 (HCoV-19) is a novel coronavirus (CoV), first discovered in Wuhan, China, in December, 2019. It is an exceptionally infectious virus that has since then spread to more than 216 countries across the world causing a form of acute pulmonary respiratory disorder named COVID-19 (coronavirus disease 2019) and was declared a pandemic by the World Health Organization (WHO) on March 11, 2020. As of August 19, 2020, SARS-CoV-2 has affected about 21.7 million people with a mortality rate of around 3.5% (World Health Organization, 2020).

Severe acute respiratory syndrome coronavirus 2 is essentially a beta-CoV possessing a positive-sense single-stranded RNA genome with a 5′ cap, a 3′ poly-A tail, and 5′- and 3′ UTRs (untranslated regions; Chen and Zhong, 2020). It is the seventh CoV known to infect humans, which includes the likes of SARS-CoV, Middle East respiratory syndrome coronavirus (MERS-CoV), HCoV-OC43, HCoV-229E, HCoV-NL63, and HCoV-HKU1 (Centers for Disease Control and Prevention, 2020). However, the origin of SARS-CoV-2 is presumed to be from bats. Andersen et al. (2020) have reported that SARS-CoV-2 has about 96% sequence similarity to RaTG13, a CoV isolated from the bat *Rhinolophus affinis*. On the other hand, cryo-EM data have shown that structurally, SARS-CoV-2 has significant similarity to the SARS-CoV (Wrapp et al., 2020). Additionally, both SARS-CoV and SARS-CoV-2 utilize the same molecule, angiotensin-converting enzyme 2 as a receptor for entry into the host cells (Samavati and Uhal, 2020). Like its predecessors SARS-CoV and MERS-CoV, to date, no vaccine or drug has proven to be successfully effective against the deadly HCoV-19 virus. Although a number of promising therapeutic candidates are in advanced stages of clinical trials, it is too early to predict their outcome.

Generally, CoVs are known to encode 16 non-structural proteins (nsps), mainly related to their RNA synthesis (Perlman and Netland, 2009). However, a unique feature of CoVs is the fact that nsp14 of CoVs codes for a special 3′–5′ exoribonuclease called ExoN. ExoN is found to be essential for RNA replication fidelity by regulating a complex RNA-dependent RNA proofreading machinery, in a manner distinct from the viral RNA–dependent RNA polymerase (RdRp), a mechanism uncommon and unprecedented in other RNA viruses (Minskaia et al., 2006). Perhaps this ExoN-mediated enhanced fidelity gives the CoVs advantage over genetic bottleneck events and prevents accumulation of deleterious mutations beyond tolerance limit, which could even lead to reduced virulence (Denison et al., 2011). This aids in persistence of infection by increasing the stability of the virus. Recently, SARS-CoV-2 nsp14 gene has shown about 95% sequence identity (Shannon et al., 2020) and 98.7% structural similarity (Gordon et al., 2020) with that of SARS-CoV. It would be interesting to check if SARS-CoV-2 possesses a functional ExoN and, if so, whether the ExoN activity has any effect on the infectivity, viability, and persistence of SARS-CoV-2 in the host. ExoN may also provide cues for understanding the viral replication, which is important for understanding the pathogenesis of the virus.

In order to successfully develop a therapeutic target for SARS-CoV-2, it is very important to understand the viral replication mechanism as well as host–virus interaction and how those may be regulated inside the host. While the development of protein-based vaccines or drug targets is still underway, it may be important to shift the focus to another group of endogenous regulatory molecules—the small non-coding RNAs (ncRNAs) such as microRNAs (miRNAs or miRs), which are known to be instrumental in regulating gene expression under various infectious disease conditions and have shown significant potential as therapeutic targets. In this review, we will be discussing the importance of miRNA-mediated gene regulation in RNA virus infections and their prospect as a therapeutic target in the ongoing SARS-CoV-2–mediated pandemic, COVID-19.

## RNA Viruses, Including CoVs, Can Encode miRNA-Like Small Regulatory RNAs

MicroRNAs, about 18–22 nucleotides long, are a class of ncRNAs that regulate gene expression under various stress, infection, or disease conditions, typically by binding to the 3′ UTR of the target mRNA by virtue of which they may either repress its translation or cause deadenylation and decay of the target mRNA (Filipowicz et al., 2008). Viruses, especially DNA viruses, are known to encode miRNAs and generally utilize the host cellular miRNA machinery for doing so (Kincaid and Sullivan, 2012). The existence of miRNAs in RNA viruses, however, was initially controversial, because they were believed to not encode miRNAs in order to avoid excision of their genomes during excision of the precursor miRNAs by the miRNA processing machinery (Varble and tenOever, 2011). Particularly, the cytoplasmic RNA viruses were thought to not express miRNAs as they may not get access to the nuclear enzyme Drosha needed for miRNA processing. However, Shi et al. (2014) showed for the first time that hepatitis A virus, a cytoplasmic RNA virus, can encode in its coding region, not one, but two novel functional miRNAs, namely, hav-miR-1-5p, and hav-miR-2-5p. This indicated that cytoplasmic RNA viruses too may express small regulatory RNAs such as miRNAs. However, miRNAs of RNA viruses do not seem to possess the canonical stem-loop structure present normally, and as such, their biogenesis and mechanism of function are not very clear (Varble and tenOever, 2011; Mishra et al., 2020). Nonetheless, significant research has been focused on identifying such altered ncRNAs during various cytoplasmic RNA virus–mediated infections, including those by the CoVs. Peng et al. (2011) had initially conducted next-generation sequencing of lung samples from each of SARS-CoV and influenza virus–infected mice strains and had shown differential expression of a number of small ncRNAs—including miRNAs, small nucleolar RNAs or snoRNAs, and non-annotated novel small RNAs—in the host cells when infected by each of the viruses. Morales et al. (2017) too performed deep sequencing of RNA from the lungs of SARS-CoV–infected mice and found three types of small viral RNAs (svRNAs), about 18–22 nucleotides long, much like miRNAs, being produced, depending on the degree of viral replication. These svRNAs were seen to modulate the host response to viral infection by regulating the production of certain proinflammatory cytokines (viz. CCL2, interleukin 6, and CXCL10).

Thus, these studies were the stepping stones that paved the way for further exploring how miRNAs may be instrumental in regulating the host–virus dynamics during RNA virus–mediated infections, particularly in the case of HCoV-mediated diseases.

## Significant Findings on miRNA-Based Gene Regulation During HCoV Infection, Including That of SARS-CoV-2

In SARS-CoV-2 infection, much like SARS-CoV, the main cause of death is ARDS or acute respiratory distress syndrome, which results from an uncontrolled systemic inflammatory response in the host in the form of a deadly antiviral “cytokine storm” (Huang et al., 2020). As the host immune response is triggered, it may lead to differential expression of both viral and host miRNAs, as mentioned previously, which in turn, may have various repercussions. Viral miRNAs may regulate different stages of their own life cycle; they may either induce or inhibit the expression of host miRNAs involved in host antiviral response pathways or regulate the turnover and function of host mRNAs related to the same. Host miRNAs, on the other hand, may regulate viral replication and may even regulate or induce the proviral host factors (Bruscella et al., 2017). Thus, the altered miRNA expression may be in favor of or against the existence and replication of the virus inside the host, depending on the host–virus dynamics during viral infection.

MicroRNAs have been reported to affect the mechanism of pathogenesis in a number of respiratory RNA viral infections. For example, in influenza virus (H1N1), miR-323, miR-491 and miR-654 cause a decrease in the expression of viral genes in the infected host by inhibiting viral replication via degradation of the transcript for a viral RNA polymerase catalytic subunit (Song et al., 2010; Izzard and Stambas, 2015; Leon-Icaza et al., 2019), whereas in Rhino viruses, miR-23b leads to downregulation of the transmembrane lipoprotein receptors used by the virus to infect host cells (Ouda et al., 2011; Tahamtan et al., 2016). However, when it comes to CoV-mediated diseases, miRNA-based gene regulation is still a relatively new and upcoming area of research. Nonetheless, there are some promising findings with respect to CoV-mediated diseases in animals. For example, miR-221-5p is seen to directly target the 3′ UTR of genomic RNA of the porcine epidemic diarrhea virus to inhibit its replication and lead to activation of the nuclear factor (NF-kβ)–mediated signaling cascade (Zheng H. et al., 2018). For HCoVs as well, some very interesting and important observations have been made, including those for SARS-CoV-2, which are highlighted as below and summarized in Table 1:

**TABLE 1:** List of important miRNAs involved in some HCoV infections.

**Human coronavirus**	**Dysregulated miRNAs**	**Target molecules/pathway**	**References**
HCoV-OC43 (N protein)	miR-9	Host NF-kβ pathway(activation)	Lai et al., 2014
SARS-CoV	[miRNAs-17*, -574-5p, and -214] (upregulation) miR-223 (downregulation) miR-98 (downregulation)	Viral replication (inhibition) Viral nucleocapsid (N) protein Viral spike (S) protein	Mallick et al., 2009
SARS-CoV-2	[miR-1307-3p miR-3613-5p] [hsa-let-7a, hsa-miR-101, hsa-miR-125a-5p, hsa-miR-126, hsa-miR-222, hsa-miR-23b, hsa-miR-378, hsa-miR-380-5 hsa-miR-98]	Viral 3′ UTR *Host protein targets*: Interferon β (IFN-β), ATP synthase F1 subunit β (ATP5B), Erb-B2 receptor tyrosine kinase 2 (ERBB2) *Viral protein targets*: Polymerase basic protein 2 (PB2) Polymerase acidic protein (PA) Non-structural protein (NS1) Nucleoprotein (NP) major capsid protein (VP1)]	Chen and Zhong, 2020; Sardar et al., 2020

1. *HCoV nucleocapsid protein (N) can directly bind to small regulatory RNAs and modulate antiviral immune response in host*:

N protein of CoVs is an RNA-binding protein that binds to the genomic RNA of the virus to form a helical capsid and plays an important role in viral replication (Wurm et al., 2001; Zúñiga et al., 2007). Lai et al. (2014) had an unexpected observation wherein they showed that upon HCoV-OC43 infection, N protein could bind to miR-9 and lead to host inflammatory response by NF-kβ activation. miR-9 is known to negatively regulate NFKB1, involved in the NF-kβ pathway (Bazzoni et al., 2009), and under stimulus and/or OC43 infection, expression of both miR-9 and NFKB1 increases. But Lai et al. observed that miR-9 cannot interact with NFKB1 as it is bound by the N protein, which eventually leads to prolonged activation of the NF-kβ pathway.

Interestingly, Cui et al. (2015) noted that coronaviruses can also utilize the N protein as a viral suppressor of RNA silencing (VSR) to overcome the antiviral response elicited by host RNAi mechanism. They showed that the coronavirus N protein could sequester siRNAs and other double-stranded RNAs, thereby preventing Dicer-mediated processing of these RNA molecules. In that way, the virus could facilitate its replication by suppressing the host RNAi-mediated antiviral response against the infection.

Thus, a viral genomic RNA-binding protein was found to be capable of binding to host miRNAs and siRNAs and modulate its inflammatory response. Because the nucleocapsid N protein is found in other HCoVs as well, it could be an important mechanism for these viruses to modulate the host immune response and may be an important area of focus while designing therapeutic antiviral strategies. It would be interesting to explore whether any such N protein-miRNA–mediated interaction is elicited in the form of host response mechanism during SARS-CoV-2 infection.

2. *Endonuclease APE1 can cleave miRNAs and other RNA components of SARS-CoV*:

APE1 is an apurinic/apyrimidinic endonuclease 1, but interestingly, Kim et al. (2010) showed that APE1 has RNA cleaving properties as well. In the absence of divalent metal ions, APE1 was seen to cleave mRNAs (c-myc, CD44), miRNAs (miR-10b, miR-21), and three RNA components of SARS-CoV, namely, Orf1b, Orf3, and spike. Thus, APE1 can potentially cleave any RNA *in vitro*. It would be interesting to see if this RNA-cleaving property of APE1 may be further explored as a tool to target specific regulatory RNAs implicated in viral infections, including that of SARS-CoV-2.

3. *SARS-CoV exploits the miRNA machinery of bronchoalveolar stem cells (BASCs) for persistent infection*:

Bronchoalveolar stem cells are one of the major cell types infected by SARS-CoV (Mallick et al., 2009; Leon-Icaza et al., 2019). Upon SARS-CoV infection, Mallick et al. observed that miRNAs-17^∗^, -574-5p, and -214 were upregulated in BASCs, which the virus utilized to suppress its own replication and bypass elimination by the host immune system. On the other hand, miR-223 and miR-98, which target nucleocapsid (N) and spike (S) proteins, respectively, were found to be downregulated, eventually leading to activation of proinflammatory cytokines during the viral infection.

4. *Computational predictions of altered miRNAs related to SARS-CoV-2 infection*:

Most of the information regarding dysregulated miRNAs during SARS-CoV-2 infection has been based on computational predictions so far. Nonetheless, these findings seem quite promising and are worth to be noted. For example, miR-1307-3p and miR-3613-5p are reported to be downregulated in lung cancer patients (Zheng Y. et al., 2018; Song et al., 2019). Interestingly, software-based predictions have shown that these miRNAs have significant binding probability to the 3′ UTR of SARS-CoV-2 as well. Moreover, tyrosine kinase inhibitors used to treat lung cancer patients was also found to upregulate miR-1307-3p (Chen and Zhong, 2020). Sardar et al. (2020) too, using *in silico* methods, have identified nine miRNAs, namely, hsa-let-7a, hsa-miR-101, hsa-miR-125a-5p, hsa-miR-126, hsa-miR-222, hsa-miR-23b, hsa-miR-378, hsa-miR-380-5, and hsa-miR-98, unique to only SARS-CoV-2 infection. The genes predicted to be targeted by these miRNAs were enlisted as interferon β (IFN-β), ATP synthase F1 subunit β (ATP5B), Erb-B2 receptor tyrosine kinase 2 (ERBB2), and some viral (influenza A and enterovirus) proteins such as polymerase basic protein 2 (PB2), Polymerase acidic protein (PA), non-structural protein (NS1), nucleoprotein (NP), and major capsid protein (VP1). These interesting findings, however, are subject to further experimental validations in *in vitro* and *in vivo* models of SARS-CoV-2. Important *in silico* comparative studies of different miRNAs dysregulated in a number of CoV infections including SARS-CoV-2 have also been done by Demirci and Adan (2020), Fulzele et al. (2020) and Identify et al. (2020)).

These observations are just the tip of the iceberg, and there is a lot of scope for exploring how miRNAs might regulate the pathogenesis of SARS-CoV-2. To begin with, it is important to perform deep sequencing to get a clear picture of the entire RNA repertoire of the virus, with special focus on the small ncRNAs. It would also be interesting to explore if SARS-CoV-2 encodes its own viral miRNAs and, if so, how those may affect the host immune response against the virus. The biogenesis and processing pathway of viral miRNAs may too withhold important information for designing future therapeutic strategies. As mentioned previously, cytoplasmic RNA viruses usually produce miRNAs in a non-canonical miRNA biogenesis pathway, probably in a Drosha- or Dicer-independent manner (Usme-Ciro et al., 2013). A type of short introns called mirtrons may be responsible for miRNA processing in a Drosha-independent pathway. Mirtrons are processed by spliceosome to produce pre-miRNAs, which are then exported via exportin-5 from the nucleus, subsequently processed by Dicer and loaded to the RISC complex for initiation of gene silencing process (Ruby et al., 2007). Alternatively, miRNA processing may also occur in a Dicer-independent manner via Simtrons (splicing-independent mirtron-like miRNAs) through a pathway that includes Drosha but excludes DGCR8, exportin-5, Dicer, and AGO-2, which are otherwise crucial for miRNA biogenesis in the canonical pathway (Havens et al., 2012). Recently, Drosha-dependent miRNA processing has been observed in certain cytoplasmic RNA viruses where, during infection, Drosha cannot get phosphorylated and, in the absence of such nuclear localization signal, the unphosphorylated Drosha is redistributed to cytoplasm where it induces non-canonical miRNA biogenesis (Shapiro et al., 2012). However, miRNA biogenesis and processing in the case of SARS-CoV-2 are unknown until now and needs to be explored. If indeed identified, they may prove to be important factors in the prognosis of SARS-CoV-2 as the factors involved in the non-canonical miRNA processing pathway of the virus are majorly different from those involved in the canonical miRNA biogenesis pathway of the host and as such can become important for specifically targeting the viral miRNAs while designing therapeutics. It may also help in bypassing viral suppression of RNAi, which mainly targets factors of the canonical miRNA processing pathway and thus prevent viral resistance against RNAi (Leonard and Schaffer, 2006).

Thus, study of regulatory ncRNAs like miRNAs demands serious research attention as it may withhold critical cues for understanding viral disease pathogenesis, how the host immune response mechanisms may be modified during viral infections, and whether it has any effect on the replication or persistence of the virus inside the host. It may be an especially important point to ponder with the view of developing successful therapeutics against SARS-CoV-2, whose infectivity has encompassed the world population.

## RNAi-Based Antiviral Therapies Targeted at miRNA-Mediated Gene Regulation Show Clinical Potential

Since the last decade or so, research has been focused on either overexpressing or downregulating miRNAs by using miRNA mimics and miRNA antagomirs (antisense miRNAs), respectively, as therapeutics against various diseases (Hanna et al., 2019). To enhance their stability, these miRNA therapeutics are usually designed in the form of locked nucleic acids or LNAs, which are chemically modified RNAs with reduced reactivity. The clinical potential of these miRNA-targeted therapeutics may be assessed from the fact that many have shown considerable promise in clinical trials for a number of diseases. For example, MRG-201/Remlarsen (LNA RNA mimic for miR-29b) is in phase II clinical trial against fibrotic diseases, whereas MRG-106/Cobomarsen (LNA antagomir against miR-155) is in clinical trials (phase II) for treating T-cell lymphomas (Bonneau et al., 2019). In recent times, miRNA inhibitors have also shown immense prospect as an antiviral therapeutic. For example, hepatitis C virus (HCV), a positive-sense single-stranded RNA virus, is known to utilize liver-specific miR-122 to positively regulate its own copy number (Jopling et al., 2005; Cox and Sullivan, 2014). Antisense inhibitor of miR-122 has proved to be effective against HCV, and currently, Santaris Pharma/Roche has developed the same under the trade name Miravirsen, which has completed phase II clinical trials in 2017 (Sarnow and Sagan, 2016). Research has also been focused on targeting viral miRNAs, instead of host miRNAs in treating viral infection–based diseases as it offers more specificity. In the case of Epstein-Barr virus, for example, antagomirs against viral miR-BART7-3p have shown positive results in mice models (Cai et al., 2015; Wang et al., 2019).

LNA-based strategy of treating antiviral infections offers various advantages, besides being highly stable. First, LNAs usually show high target specificity and lower off-target effects compared to other RNAi-based strategies. Second, they are not known to interfere with other therapeutics and may in fact be used in a combinatorial manner for increased efficacy of treatment.

## Delivery Strategies for Successful RNAi-Based Therapeutics Against COVID-19

An effective RNAi-based therapeutics targeting miRNAs also requires a suitable and efficient delivery system. *In vivo* delivery of chemically modified miRNA or anti-miRNA oligonucleotides may be obtained via two main carrier systems: viral vectors and non-viral carriers (Baumann and Winkler, 2014; Fu et al., 2019).

Viral vectors are vectors with a viral backbone, modified to transfer gene of interest to the target organs/tissues with high efficiency for long-term gene expression/repression. The commonly used viral vectors for miRNA delivery are as follows:

1. *Adenoviral vectors* (*AdVs*), derived from the Adenoviridae family, may be used for miRNA delivery across various tissues and organs. For example, in the case of hepatitis B virus, delivery of pri-miR-122/5, pri-miR-31/5, and pri-miR-31/5-8-9 by AdVs led to short-term inhibition of viral replication in the liver tissue (Mowa et al., 2014). However, these vectors are found to elicit strong immune responses in the host, which largely restricts its use as a gene transfer vehicle.
2. *Adeno-associated viral vectors* are derived from the Parvoviridae family and lead to sustained gene expression *in vivo* (Miyazaki et al., 2012).
3. *Retroviral vectors* (*RVs*) are RNA viral vectors that, when they enter the host cells, produce double-stranded DNA by the reverse transcriptase enzyme and integrate into the host genome, thus offering sustained expression of the gene of interest. RVs are used commonly to deliver miRNAs (Ramanujam et al., 2016).
4. *Lentiviral vectors* (*LVs*) too are RNA virus vectors, and like RVs, they too integrate into the host genome, thus offering persistent expression of the inserted gene. For example, miR-133b was delivered by LVs to ameliorate spinal cord injury in mice (Theis et al., 2017).

Although the viral vectors are usually modified for reduced immunogenicity, they may still show significant antigenic reactions and cytotoxicity in some cases and are thus nowadays replaced with non-viral vectors. Some non-viral carriers commonly implicated and reported to be used in delivery of miRNA based therapeutics are as follows:

1. *Inorganic material–based systems*: They include gold nanoparticles, which when modified by attaching thiol or amino groups to their surface become suitable for transferring miRNAs *in vivo* (Jia et al., 2017).
2. *Lipid-based nanovectors*: Cationic lipids are amphiphilic molecules possessing both hydrophilic (head) and hydrophobic (tail) parts, suitable for delivering oligonucleotides *in vivo*. They may be conjugated with non-ionic hydrophilic polymers such as polyethylene glycol (PEG) for increased delivery efficiency and stability within the tissue/organ (Endo-Takahashi et al., 2015).
3. *Cell-derived membrane vesicles*: Extracellular vesicles (EVs) are membrane-bound vesicles released by the cells, usually for cell-to-cell communication. Depending on size, EVs may be broadly classified into exosomes (40–120 nm), microvesicles (100–1,000 nm), and apoptotic bodies (50–5,000 nm). These EVs have the advantage of low cytotoxicity, negligible immunogenicity, and high delivery efficacy and thus show great potential for delivery of miRNA based therapeutics (Zhou et al., 2018).
4. *Three-dimensional (3D) scaffold–based systems*: 3D biomaterial scaffolds such as hydrogels and electrospun fibers when conjugated with PEG have proven to lead to stabilized and sustained RNAi delivery (Nguyen et al., 2018).

However, the greatest advantage of RNAi in respiratory diseases such as COVID-19 is the fact that administration of miRNAs directly to the lung tissue may be achieved by inhalation. Oligonucleotides are water-soluble and may be aerosolized to penetrate both upper and lower respiratory tracts. For example, live attenuated viral vaccine expressing miR-93 when administered intranasally could successfully confer protection against viral infections (DeVincenzo, 2012; Li et al., 2015; Leon-Icaza et al., 2019). In another scenario, intranasal administration of siRNA (ALN-RSV01) against respiratory syncytial virus (RSV) nucleocapsid protein has been successful in phase II clinical trials against RSV-induced respiratory disease (Dyawanapelly et al., 2014).

Thus, depending on the nature of the RNAi-based therapeutics, any of the previously mentioned delivery mechanisms may be utilized for effective RNAi-based therapy against COVID-19.

## Significance of RNAi as a Therapeutic Strategy to Treat SARS-CoV-2–Mediated Infection

Designing an appropriate therapeutic against SARS-CoV-2 needs to consider certain factors. First, SARS-CoV-2 is reported to be evolving into various mutant subtypes and strains (Mercatelli and Giorgi, 2020; Osório and Correia-Neves, 2020; Tang et al., 2020). Second, so far, SARS-CoV-2 is known to cause acute infections, lasting generally for about 2 to 4 weeks. To address these concerns, miRNA-based therapies may be useful as miRNAs can evolve fairly fast and target new mRNA transcripts, which give them a certain functional flexibility. Additionally, instead of targeting a single gene, a combinatorial RNAi targeting multiple conserved genes commonly implicated across a broad range of SARS-CoV-2 strains may be more effective in combating the disease and may prevent viral escape or development of viral resistance against RNAi. Combinatorial RNAi has also shown potential against other acute viral infections such as influenza A (Estrin et al., 2018). However, critical considerations must be made to minimize cytotoxicity and off-target effects of RNAi while designing such therapeutic strategies. Second, RNAi appears to be more appropriate in targeting acute infections where consecutive administration of synthetic oligonucleotides only a limited number of times may prove to be sufficient in suppressing the infection, as compared to persistent infections where a stable expression system is necessary to elicit a prolonged effect of the therapeutic (Ketzinel-Gilad et al., 2006). Moreover, RNAi strategy may be designed to offer a transient or reversible effect which may be advantageous since long-term silencing of certain genes may prove to be toxic to the cell. Most importantly, most acute infections induce a “cytokine storm” as previously mentioned, leading to activation of the host immune responses to combat the viral infection. In such a scenario, modulation of antiviral genes within a short span of time as offered by the miRNA machinery may not only enhance the host immune response but also provide a robust and quick means of eliminating the virus, which may be especially important in a pandemic situation.

## Future Prospects of Regulatory ncRNAs in Combating COVID-19

From the above discussion, it is quite evident that regulatory ncRNAs, particularly miRNAs, are emerging as important players in regulating virus-mediated infection and subsequent disease conditions. miRNA-mediated gene regulation is unique, because a single miRNA may have multiple mRNA targets, or a single mRNA may be targeted by multiple miRNAs. This pleotropic nature of miRNAs makes it a suitable therapeutic candidate. Additionally, because miRNAs have an endogenous origin and are processed utilizing the host cellular machinery, there is less fear of eliciting immunogenic reaction against them.

However, recent reports suggest that CRISPR-Cas13–based gene editing may be a potential alternative therapeutic strategy against viral infections including that by SARS-CoV-2 (Freije et al., 2019). Particularly, Abbott et al. (2020) developed a CRISPR-Cas13d–based intervention strategy named PAC-MAN (prophylactic antiviral CRISPR in human cells) to target conserved viral regions across different strains of each of SARS-CoV-2 and H1N1 viruses, with the view of developing a pan-coronavirus inhibition therapeutic strategy. Although promising, these CRISPR-based strategies need to cross multiple checkpoints like minimizing off-target effects, devising effective *in vivo* delivery methods, and assessment of probable immunogenic and cytotoxic effects must be done before being considered as suitable clinical intervention options, which might take a good few years. Thus, in the rising emergency of COVID-19, RNAi seems to be more suitable as it is a comparatively time-, labor-, and cost-effective therapeutic strategy.

To add to that, miRNAs, including viral miRNAs are clinically relevant as biomarkers because they may be secreted in body fluids such as blood, saliva, urine, etc. For example, viral miR-VP-3p can be detected in the sera of Ebola virus–infected patients (Chen et al., 2016), whereas miR-US4-1 is found in the sera of patients with hepatitis B virus receiving IFN-a treatment (Pan et al., 2016). The clinicaltrials.gov database lists completed clinical studies for miRNA biomarkers, which even includes advanced (phase IV) clinical trials.

In the past, no vaccine or drug has been proven effective against HCoVs, including the likes of SARS-CoV or MERS-CoV, which too were responsible for pandemic respiratory diseases in 2002 and 2012, respectively. Although clinical trials of potential vaccines and drug targets are ongoing against COVID-19, it is too early to predict how useful they are eventually going to be in eliminating SARS-CoV-2, which is reported to be mutating and evolving at a considerable rate. In such a scenario, identification of altered or dysregulated miRNAs during SARS-CoV-2 infection may prove instrumental in designing RNAi-based therapeutics for combating COVID-19 and may open up new avenues for developing biomarkers against this deadly pandemic in the future.

## Author Contributions

PB conceived and wrote the manuscript. SB supervised and reviewed the article. Both authors contributed to the article and approved the submitted version.

## Conflict of Interest

The authors declare that the research was conducted in the absence of any commercial or financial relationships that could be construed as a potential conflict of interest.
